# In Vivo Assessment of Hypoxia Levels in Pancreatic Tumors Using a Dual-Modality Ultrasound/Photoacoustic Imaging System

**DOI:** 10.3390/mi12060668

**Published:** 2021-06-07

**Authors:** Yuhling Wang, De-Fu Jhang, Chia-Hua Tsai, Nai-Jung Chiang, Chia-Hui Tsao, Chiung-Cheng Chuang, Li-Tzong Chen, Wun-Shaing Wayne Chang, Lun-De Liao

**Affiliations:** 1 Institute of Biomedical Engineering and Nanomedicine, National Health Research Institutes, No. 35, Keyan Rd., Zhunan Township, Miaoli 35053, Taiwan; yuhlingwang@nhri.edu.tw (Y.W.); y217834@gmail.com (D.-F.J.); vanessatsai@nhri.edu.tw (C.-H.T.); tsaochiahui@nhri.edu.tw (C.-H.T.); 2 Department of Biomedical Engineering, College of Engineering, Chung Yuan Christian University, No. 200, Chung Pei Road, Chung Li District, Taoyuan 32023, Taiwan; cheng965@cycu.edu.tw; 3 National Institute of Cancer Research, National Health Research Institutes, No. 35, Keyan Rd., Zhunan Township, Miaoli 35053, Taiwan; njchiang@nhri.edu.tw (N.-J.C.); leochen@nhri.edu.tw (L.-T.C.); 4 Department of Oncology, National Cheng Kung University Hospital, Tainan 70403, Taiwan; 5 Kaohsiung Medical University Hospital, Kaohsiung Medical University, No. 100, Shih-Chuan 1st Road, Sanmin Dist., Kaohsiung 80708, Taiwan

**Keywords:** pancreatic tumor, photoacoustic (PA), ultrasound (US), hypoxia, hemoglobin oxygenation saturation

## Abstract

Noninvasive anatomical and functional imaging has become an essential tool to evaluate tissue oxygen saturation dynamics in preclinical or clinical studies of hypoxia. Our dual-wavelength technique for photoacoustic (PA) imaging based on the differential absorbance spectrum of oxyhemoglobin (oxy-Hb) and deoxyhemoglobin (deoxy-Hb) can quantify tissue oxygen saturation using the intrinsic contrast property. PA imaging of tissue oxygen saturation can be used to monitor tumor-related hypoxia, which is a particularly relevant functional parameter of the tumor microenvironment that has a strong influence on tumor aggressiveness. The simultaneous acquisition of anatomical and functional information using dual-modality ultrasound (US) and PA imaging technology enhances the preclinical applicability of the method. Here, the developed dual-modality US/PA system was used to measure relative tissue oxygenation using the dual-wavelength technique. Tissue oxygen saturation was quantified in a pancreatic tumor mouse model. The differences in tissue oxygenation were detected by comparing pancreatic samples from normal and tumor-bearing mice at various time points after implantation. The use of an in vivo pancreatic tumor model revealed changes in hypoxia at various stages of tumor growth. The US/PA imaging data positively correlated with the results of immunohistochemical staining for hypoxia. Thus, our dual-modality US/PA imaging system can be used to reliably assess and monitor hypoxia in pancreatic tumor mouse models. These findings enable the use of a combination of US and PA imaging to acquire anatomical and functional information on tumor growth and to evaluate treatment responses in longitudinal preclinical studies.

## 1. Introduction

Hypoxia is a condition present in a wide range of diseases, including stroke [1], pneumonia, cancer [2], sleep apnea [3], etc. Recently, optical imaging techniques, such as diffusion optical imaging (DOI) [4], were used to assess in vivo changes in tissue oxygenation. DOI can be used to directly detect hemoglobin oxygen saturation (SO_2_) signals; however, DOI has insufficient spatial resolution and thus cannot delineate the fine details of specific structures due to the diffusive nature of light in biological tissues [4]. Photoacoustic (PA) imaging is a rapidly developing technology based on the optical excitation of the molecules by visible or near-infrared (NIR) range pulsed lasers, resulting in a thermoelastic effect of PA signals [4]. The expansion and contraction caused by the absorption of laser energy generates ultrasound (US) waves that can be detected and reconstructed by the traditional US technique. By utilizing intrinsic optical absorption along with US detection, PA imaging can provide multiscale resolution and deep tissue penetration [4]. Depending on the intrinsic biological optical contrast agent (i.e., blood or melanin), the PA imaging technique offers anatomical and functional information about normal and abnormal tissues and biological phenomena, such as angiogenesis and changes in SO_2_ and total hemoglobin concentrations [5]. Additionally, some advantages of PA are similar to the advantages of US and optical imaging, such as safety and high spatial resolution [4].

Previously, we developed a functional PA microscopy (fPAM) technique that can preclinically evaluate the changes in functional cerebral blood volume (CBV) and SO_2_ in the rat cortex in studies of ischemic stroke [6,7,8]. The dual-wavelength PA technique uses hemoglobin as an intrinsic contrast agent and thus does not require an exogenous contrast agent for blood volume and SO_2_ imaging [9]. Numerous fPAM applications, especially in preclinical studies, have been gradually developed in recent years [10,11,12,13].

In addition to the characterization of ischemic stroke [13], our fPAM system can also be applied to monitoring SO_2_ in pancreatic cancer. Pancreatic cancer is a lethal disease that has a limited response to cytotoxic chemoradiotherapy [14,15] and even newer immunotherapies [2,3]. The limited success of immunotherapy in pancreatic cancer is likely due to the numerous immunosuppressive pathways upregulated in the hypoxic tumor microenvironment [16,17]. Hypoxia in pancreatic tumors is heterogeneous and exacerbates the complex immunosuppressive environment [18]. Hypoxia occurs in most solid tumors because the abnormal vasculature cannot provide adequate oxygen delivery demanded by rapidly proliferating cancer cells [19]. Because tumor hypoxia is a major impediment to effective cancer therapy [20,21], it is important to assess the dynamics of hypoxia in pancreatic tumors in real-time at various tumor stages or in drug treatment studies in preclinical models.

Current common methods for assessing tumor hypoxia in preclinical models include the use of oxygen electrodes [22], imaging of hypoxia biomarkers [23], blood oxygen level-dependent (BOLD) functional magnetic resonance imaging (fMRI) [24,25], and positron emission tomography (PET) [26]. Each technique evaluates various aspects of the hypoxic microenvironment because different aspects provide specific information on hypoxia [27,28]. BOLD MRI and fMRI techniques are noninvasive and can be used for multiparametric measurements, including blood oxygenation, blood flow, blood volume, and oxygen metabolism [29,30]. BOLD MRI or fMRI techniques do not have tissue depth limitations; however, they cannot be used to investigate transient functional hemodynamic responses in the blood vessels with unambiguous resolution [25,30]. In addition to fMRI, ultrasound imaging is being actively developed [31]. Excellent reviews of these technologies are available in the literature [32,33].

In this study, we aimed to address the feasibility of the investigation of real-time pancreatic tumor oxygenation dynamics using a label-free approach by three-dimensional (3D) PA imaging in parallel with conventional B-mode US imaging. We initially tested our PA imaging system using blood vessel phantoms in vitro. Then, we tested our system by imaging the skin vasculature of a rat in vivo. To assess the ability of our PA imaging system to quantify tissue SO_2_, we performed a controlled hypoxia study in which hypoxia was induced in vivo through the inhalation of a gas mixture routinely used for anesthesia. After the controlled hypoxia study, we imaged hypoxia in orthotopic pancreatic tumors in vivo. We provided evidence that this combined system can be used to reliably assess the differences in tissue oxygenation between control and tumor-bearing mice using dual-wavelength PA technique to provide structural information at a proper resolution. Immunohistochemical (IHC) staining for hypoxia using pimonidazole confirmed the hypoxia dynamics detected using the developed PA system with the dual-wavelength technique.

## 2. Materials and Methods

### 2.1. Dual-Modality US/PA Imaging System

**Figure 1:**
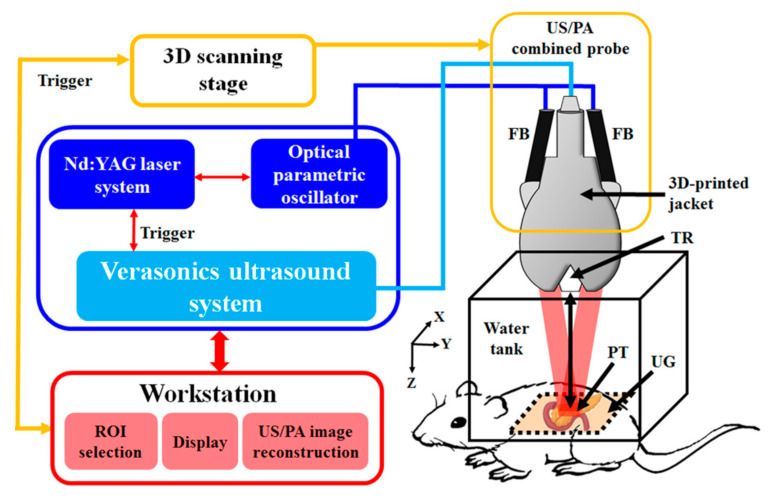
Schematic diagram of the developed dual-modality US/PA imaging system. Using the photoacoustic effect, US waves were generated by delivering light generated by a laser to the imaging target with a pulse repetition rate of 20 Hz using fiber bundles. The US waves were then detected by a 17.5-MHz transducer and processed through a Verasonics ultrasound system. The developed PA probe includes a removable fiber bundle-based illumination system (FB), a single US transducer (TR), and a customized jacket. Two rectangular parts constituted the output end of the fiber bundle, and the input end was circular in shape. ROI, region of interest; PA, photoacoustic; US, ultrasound; TR, ultrasound transducer; FB, fiber bundle; PT, pancreatic tumor; and UG, ultrasound gel.

A diagram of the developed dual-modality US/PA imaging system is shown in Figure 1, including the details of the system block design. The system consisted of a high-frequency 18.5-MHz US transducer (L22-14v, Verasonics, Inc., Kirkland, WA, USA) connected to a 128-channel Verasonics high-frequency ultrasound platform (Vantage 128, Verasonics, Inc., Kirkland, WA, USA) for data acquisition. The US transducer had a -6 dB fractional bandwidth of 67%. For PA imaging, a compact excitation Nd:YAG laser system was used with an integrated tunable optical parametric oscillator (OPO) system (SpitLight 600 OPO, InnoLas Laser GmbH, Krailling, Germany). The OPO generated approximately 7-ns-long pulses with a repetition rate of 20 Hz and tunable wavelengths from 680 to 2400 nm. A customized fiber bundle-based illumination system was used to deliver the laser energy to the imaging target from the laser output. This fiber bundle-based illumination system was designed to be 1.5-m long and contained approximately 21,000 leaded glass fibers 50 μm in thickness with a high numerical aperture (NA). Each fiber had a core diameter of 47 μm (50 μm with cladding) and an NA of 0.66. The fiber efficiency was estimated to be approximately 60–70% based on the measurements of the input and output energy at the ends of the fiber bundle-based illumination system [9]. The axial resolution was calculated as the full width at half maximum of each Gaussian function and was previously measured to be 124 ± 31 μm for our PA imaging system [4]. To image in the PA mode, the laser excitation needed to be synchronized with US data acquisition. Synchronization was performed using custom-developed software written in MATLAB (R2007a, MathWorks, Natick, MA, USA). The acoustic waves were received, reconstructed, and displayed on the computer screen at a frame rate of 20 frames per second. The American National Standards Institute (ANSI) safety limit is 20 mJ/cm^2^, and the incident energy density on the sample surface during PA imaging was estimated to be approximately 12 mJ/cm^2^, which is within the safety limit [29,34].

A customized, precision 3D translation stage with motorized x-, y-, and z-axes was used to control the transducer to obtain A-scan, B-scan (i.e., two-dimensional; one axis is the lateral scanning distance, and the other axis is the imaging depth), and C-scan (i.e., three-dimensional) images [9]. Both the phantoms and probe were immersed in a water tank for in vitro imaging. For in vivo imaging, the PA probe was immersed in an acrylic water tank with a rectangular cutout at the bottom serving as an imaging window. The cutout was sealed with a thin polyethylene film 15-μm thick. US gel (POC Medical, Inc., Zhongli City, Taiwan) or an agarose pad was then used to provide a coupling interface between the imaging window and the animal [6].

### 2.2. In Vitro Performance of the US/PA Imaging System

For in vitro testing of the US/PA imaging system, 6% Intralipid (optical absorption coefficient of 0.01 cm^−1^ and optical scattering coefficient of 400 cm^−1^; Sigma-Aldrich, St. Louis, MO, USA) was mixed in water to mimic the optical diffusion encountered in the tissues during in vivo imaging [30]. Tube phantoms containing blue or red ink to mimic oxygenated and deoxygenated blood were then placed in the Intralipid mixture for imaging. B-scan images of the tubes were obtained at the focal zone of the transducer (10 mm in depth from the transducer surface) using laser excitation at 750 nm (strong absorption for red ink) and 850 nm (strong absorption for blue ink) [9]. C-scan images composed of serial cross-sectional images of the tubes were also obtained at a scanning range of 6 mm with 0.05 mm step size.

### 2.3. In Vivo PA Imaging of Skin Vasculature

The skin vasculature was imaged on the back of male Wistar rats (National Laboratory Animal Center, Taiwan) weighing 300–350 g. All animals were housed in a controlled room (temperature, 24 ± 1 °C; relative humidity, 50–60%) with a 12-h light/dark schedule (light cycle beginning at 7 am) and free access to food and water [12,35]. All experimental procedures, animal care protocols, and protocols requiring ethical oversight were performed in accordance with the guidelines approved by the Institutional Animal Care and Use Committee (IACUC) of the National Health Research Institute (approved protocol number: NHRI-IACUC-107100).

Before imaging the rats, the hair was removed using a hair removal cream (Stellar Beauty Biotech Co., Ltd., Keelung City, Taiwan). The animals were anesthetized by inhalation of 1–3% isoflurane (Panion & BF Biotech, Inc., Taipei City, Taiwan) in oxygen [12]. The transducer was set as described previously, including an acrylic water tank, an imaging window, and US gel or an agarose pad as a coupling interface with the rat skin. Body temperature was maintained at 37 °C using a heating pad throughout the experiment. Arterial blood SO_2_ and heart rate were monitored throughout the experiment using the MouseOX Plus small animal vital signs monitoring system (Starr Life Sciences Corp., Oakmont, PA, USA). B-scan images were obtained at 750 nm due to the strong absorption by deoxyhemoglobin at that wavelength [9].

### 2.4. Use of PA Imaging to Monitor Rat Hindlimb Tissue Oxygenation during the Controlled Hypoxia Study

Research manuscripts reporting large datasets that are deposited in a publicly available database should specify where the data were deposited and provide the relevant accession numbers. If the accession numbers have not yet been obtained at the time of submission, please state that they will be provided during review. They must be provided prior to publication.

For the controlled hypoxia study, rats (n = 5) were anesthetized with 1.5–2% isoflurane in oxygen, and fur was removed from a hindlimb using hair removal cream. The gas mixture supplying the isoflurane vapor (RWD Life Science, San Diego, CA, USA) was alternated between pure oxygen (100% O_2_) to induce hyperoxia and carbogen (5% O_2_, 5% CO_2_, and 90% N_2_) to induce hypoxia over 5-min periods [36]. A pulse oximeter (MouseOX Plus, Starr Life Sciences Corp., Oakmont, PA, USA) was clipped onto the paw of the scanned limb to verify body SO_2_ [36]. For PA imaging, a deoxyhemoglobin-dominant absorption wavelength (*λ* = 750 nm) and an oxyhemoglobin-dominant absorption wavelength (*λ* = 850 nm) were used to acquire the data [6,12]. The following equations were used to calculate SO_2_ [6,8,9,12]:

(1) $$\mu_{a}^{\lambda }=\varepsilon_{HbO}^{\lambda }\left[HbO\right]+\varepsilon_{Hb}^{\lambda }\left[Hb\right]$$

where $\varepsilon_{Hb}^{\lambda }\;$and $\varepsilon_{HbO}^{\lambda }$ are the molar extinction coefficients, [*Hb*] and [*HbO*] are the concentrations of deoxy- and oxyhemoglobin, respectively, and $\mu_{a}^{\lambda }$ is the optical absorption coefficient [6,34]. The optical absorption coefficient is related to acoustic pressure through the following equation:

(2) $$\mu_{a}^{\lambda }=\frac{P}{\Gamma F}$$

where Γ is the Grüneisen coefficient [37], *P* is the acoustic pressure, and *F* is the optical fluence [5]. Laser energy was monitored using a built-in energy monitor in the OPO system, and optical fluence was assumed to be uniform. [*Hb*] and [*HbO*] can then be used to calculate *SO*_2_ using the following equation:

(3) $$SO_{2}=\frac{\left[HbO\right]}{\left[HbO\right]+\left[Hb\right]}$$

Statistical significance was assessed using a *t*-test with significance defined as *p* < 0.05 [7,25,32].

### 2.5. Mouse Pan02 Cells

The mouse Pan02 cell line was a gift from a collaborator at the National Health Research Institutes of Taiwan (Dr. Wun-Shaing Wayne Chang). The cells were maintained in Dulbecco’s modified Eagle medium (Thermo Fisher Scientific, Inc., Waltham, MA, USA) supplemented with 10% fetal bovine serum and in an incubator at 37 °C and a constant humidity in 5% CO_2_. A 0.05% trypsin-ethylenediaminetetraacetic acid (EDTA) solution (Thermo Fisher Scientific, Inc., Waltham, MA, USA) was used to detach the cells from the culture dish during passage.

### 2.6. Orthotopic Implantation of Pan02 Cells

Pan02 cells were orthotopically implanted in the pancreas of five-week-old C57B1/6 mice (National Laboratory Animal Center, Taipei City, Taiwan). All experimental procedures and animal care protocols were performed in accordance with the guidelines approved by the IACUC of the National Health Research Institute (approved protocol number: NHRI-IACUC-107113-A).

For surgery, the mice were anesthetized by the inhalation of 1–2% isoflurane in oxygen. Carprofen (5 mg/kg, Zoetis, Inc., Taipei City, Taiwan) was injected subcutaneously for analgesia. Hair around the surgical site was shaved, and the pancreas was exteriorized through a small incision in the left flank. Pan02 cells (0.5 × 10^6^) suspended in 50 μL of phosphate-buffered solution (PBS) were injected into the tail of the pancreas using a 28-gauge needle and a syringe [38]. The pancreas was then tucked back into the abdominal cavity, and the incision was closed using wound clips and size 3-0 nylon suture (UNIK Surgical Sutures Mfg. Co., Ltd., New Taipei City, Taiwan).

### 2.7. In Vivo Pancreatic Tumor Hypoxia Imaging

Normal and tumor-bearing mice were imaged on various days after tumor cell implantation. The mice were anesthetized by the inhalation of 1–2% isoflurane in oxygen and imaged through an acrylic water tank. Laser excitation wavelengths of 750 and 850 nm were used to acquire C-scan PA images for SO_2_ evaluation [6]. The scanning range and step size were 10 mm and 0.05 mm, respectively. To accurately identify the regions of interest (ROIs), US C-scan images were acquired and overlaid with PA images [6].

### 2.8. IHC Staining for Tumor Hypoxia

Pimonidazole (Hypoxyprobe, Inc., Burlington, MA, USA) was injected intraperitoneally at the dose of 60 mg/kg body weight. The mice were sacrificed 1 h after the injection, and the tumor tissue was flash-frozen in Surgipath FSC 22 clear frozen section compound (Leica Biosystems Richmond, Inc., Richmond, IL, USA). The tissue was cryosectioned at a thickness of 7 μm and fixed in ice-cold acetone (Sigma-Aldrich, St. Louis, MO, USA) for 10 min. The sections were incubated with FITC-Mab1 (clone 4.3.11.3; Hypoxyprobe, Inc., Burlington, MA, USA) overnight at 4 °C to stain for pimonidazole [39]. The slides were then mounted in Fluoroshield mounting medium with DAPI (Sigma-Aldrich, St. Louis, MO, USA) and stored at 4 °C. Images of three random fields of view (FOVs) were acquired for each sample using an upright fluorescence microscope (DM2500; Leica Microsystems, Wetzlar, Germany) and a color CCD (DP73; Olympus, MA, USA). ImageJ software was used to quantify the mean fluorescence intensity (MFI) [39], and statistical analysis was performed using one-way analysis of variance (ANOVA) with Tukey’s multiple comparison test in GraphPad Prism 6 software (GraphPad Software, San Diego, CA, USA).

## 3. Results

### 3.1. In Vitro Performance of the US/PA Imaging System

**Figure 2:**
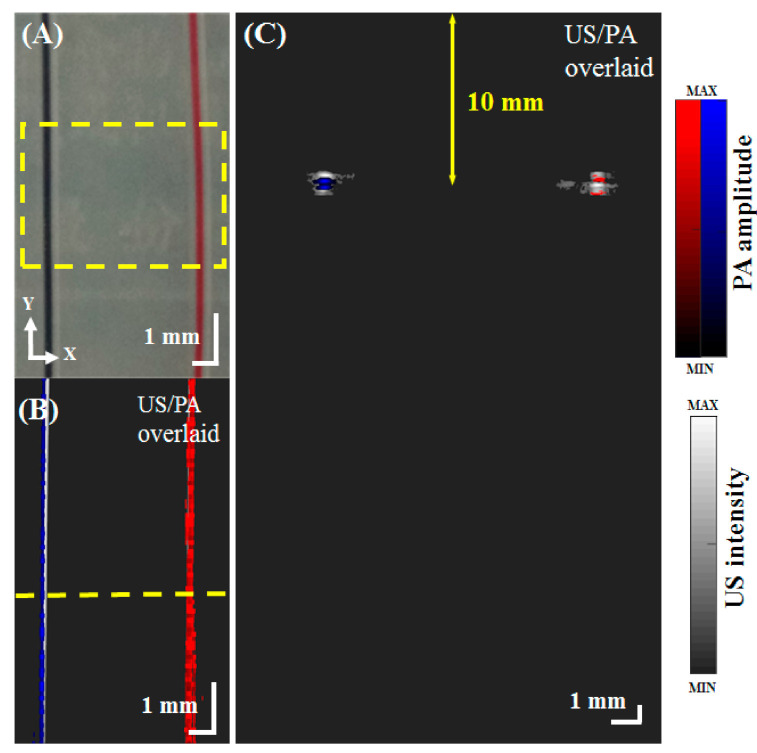
US/PA images of vessel phantoms. (**A**) Image of the tube phantoms filled with red and blue ink to mimic oxygenated and deoxygenated blood. The phantoms were imaged using US/PA within the yellow dashed rectangle (3 mm × 6 mm). (**B**) Overlaid US/PA MAP image (PA850/PA750) of the tube phantoms filled with red and blue ink. The yellow dashed line marks the location of the cross-sectional US/PA B-scan shown in Figure 2C. (**C**) Overlaid US/PA B-scan image of the phantoms at an imaging depth of 10 mm. PA, photoacoustic; US, ultrasound; PA750, PA signal at an excitation wavelength of 750 nm; PA850, PA signal at an excitation wavelength of 850 nm; and MAP, maximum amplitude projection.

To test the ability of the developed PA system to image blood vessels, tube phantoms (Figure 2A) were filled with red and blue ink to mimic oxygenated and deoxygenated blood, respectively. The region marked by a yellow-dashed rectangle was imaged using US/PA. Maximum amplitude projections (MAPs) of a C-scan image composed of serial cross-sectional images of the tubes are shown in Figure 2B. The US, PA and overlaid US/PA MAP images of the vascular phantoms are shown in Figure 2B. The yellow-dashed line marks the location of the cross-sectional scan shown in Figure 2C. At 750 nm, the PA signal of the blue ink was dominant. Because the signal intensity of both ink colors was similar at 850 nm, the proportional difference (PARed = PA850/PA750) between the two excitation wavelengths was calculated to obtain the red ink signal [4]. The resulting overlaid images shown in Figure 2B,C show the optical absorption characteristics of the blood vessel phantoms and provide anatomical and functional information [9].

### 3.2. In Vivo PA Imaging of Skin Vasculature

**Figure 3:**
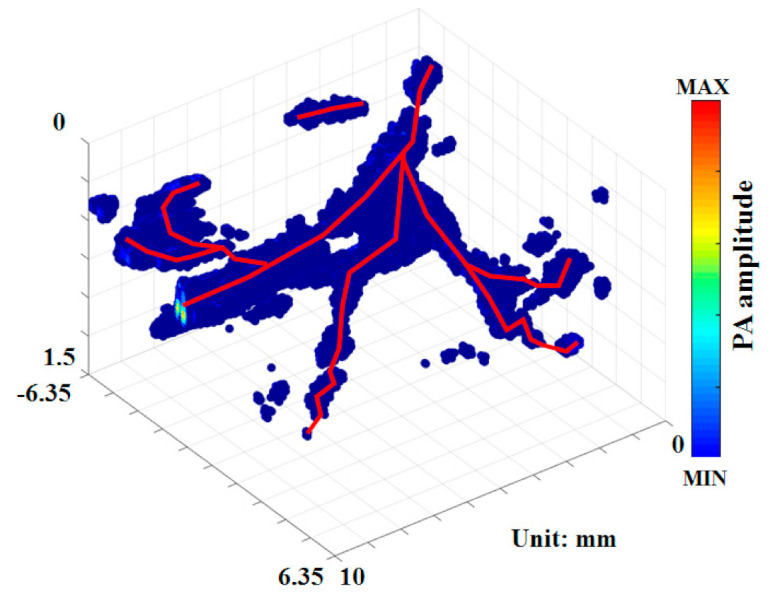
PA images of blood vessels acquired by imaging the skin on the back of a rat. Laser excitation at 750 nm was used to acquire in vivo PA C-scan images. The trends of blood vessels in the skin are indicated by solid red lines.

A PA C-scan (3D) image of the skin vasculature on the back of a rat obtained at a wavelength of 750 nm is shown in Figure 3. MAP matrices containing the peak amplitudes of the PA signals corresponding to absorbing structures on a pixel-by-pixel basis were used to plot the figure [29]. The red lines indicate the position of blood vessels. An area of 12.7 × 10 mm with a depth of 1.5 mm was scanned at a 50-µm step size.

### 3.3. In Vivo PA Monitoring of Rat Hindlimb Tissue Oxygenation during the Hypoxia Challenge

Many methods have been used to assess hypoxia. The linear model uses multi-wavelength PA signals for linear unmixing of oxyhemoglobin and deoxyhemoglobin without compensating for local fluence. Although mathematically simple and easy to implement in most photoacoustic tomography systems, the model is inaccurate at depths exceeding 1 mm for optical fluence that is independent of wavelength [40,41,42]. Optical transport models based on Beer’s law and diffusion finite-element method can be used to estimate optical fluence distribution in tissue. This model takes into account wavelength-dependent light attenuation. At depths of approximately 10 mm, this model is more accurate than linear models. However, it is not suitable for complex tissue types, such as brain and tumors [42,43]. The diffuse optical tomography method mainly evaluates the optical properties of tissue and local optical fluence to correct for the inhomogeneity of the optical fluence when imaging at larger depths (12 mm). Diffuse optical tomography is a low-resolution imaging modality, and the optical fluence compensation lacks spatial accuracy [44]. In this study, PA signals from different wavelengths were used to quantify the relative hypoxia. The equation used in this study combines molar extinction and optical absorption coefficient and is less sensitive to wavelength associated fluctuations in local optical fluence.

**Figure 4:**
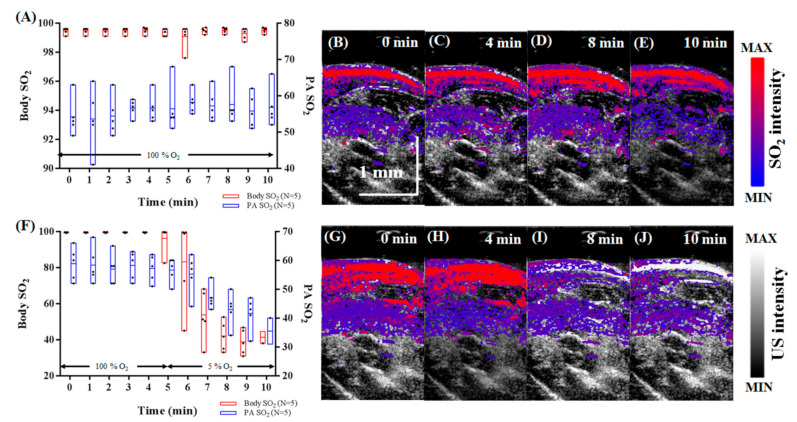
Dynamic changes in SO_2_ during a controlled hypoxia challenge. (**A**) Changes in body and PA SO_2_ as inhaled gas was maintained at 100% O_2_. (**B**–**E**) Representative PA images collected over 10 min showing tissue oxygen distribution in the hindlimb were overlaid with US images. (**F**) Changes in body and PA SO_2_ as inhaled gas was switched from 100% O_2_ to carbogen at the 5 min mark to induce hypoxia. (**G**–**J**) Representative PA/US images collected during hypoxia challenge (N = 5; * *p* < 0.05).

The results for the controlled hypoxia challenge are shown in Figure 4. For Figure 4A–E, the inhaled gas was maintained at 100% O_2_ for 10 min. In Figure 4A, the body SO_2_ measured using pulse oximetry and PA SO_2_ remained at an average of 99.41% and 56.12%, respectively. As expected, there was no significant change in the tissue oxygen level [36]. The PA SO_2_ images (Figure 4B–E) collected at 0, 4, 8, and 10 min also showed no changes in SO_2_. For Figure 4F–J, the inhaled gas was maintained at 100% O_2_ for 5 min before switching to the carbogen mixture for another 5 min to induce hypoxia. Figure 4F shows a significant decrease in both body and PA SO_2_. The body SO_2_ was significantly decreased from an average of 99.7% to 38.74%, whereas the PA SO_2_ was decreased from an average of 58.8% to 35.5%. The PA SO_2_ images shown in Figure 4G–J indicate that the oxygenated area (red) decreased after the switch to carbogen, while the ischemic area (blue) increased. These results demonstrate that the assessment of tissue SO_2_ using our PA imaging system reflects the changes in body SO_2_ measured using pulse oximetry during hypoxia challenge. A study by Xing et al. showed that changes in hemoglobin oxygen saturation and carboxyhemoglobin saturation could be observed in blood vessels using PA microscopy during carbon monoxide challenge [45]. The resolution of our US/PA imaging system does not allow for observation of single blood vessels but allows for monitoring of larger areas of tissue. After evaluation of hypoxia in a controlled in vivo setting, our next step was to measure hypoxia in the pancreatic tissue of normal and tumor-bearing mice 9.

### 3.4. In Vivo Imaging of the Changes in Pancreatic Tissue Oxygenation in Normal vs. Tumor-Bearing Mice

**Figure 5:**
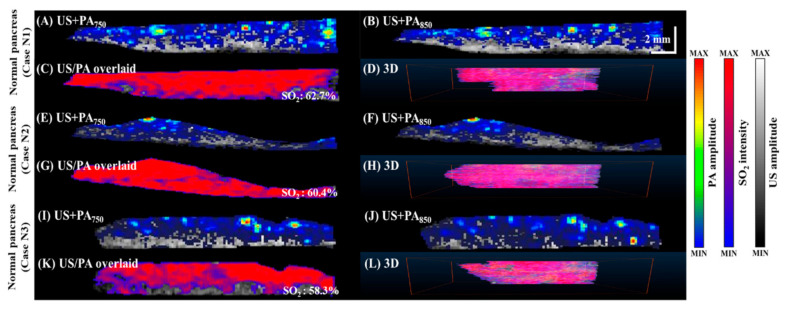
Relative SO_2_ levels in normal pancreatic regions in three different mice. SO_2_ was calculated as the percentage of oxygenated hemoglobin relative to total hemoglobin based on dual-wavelength PA imaging at 750 and 850 nm. (**A**,**E**,**I**) PA images acquired at 750 nm overlaid with grayscale US B-mode images. (**B**,**F**,**J**) PA images acquired at 850 nm overlaid with grayscale US B-mode images. (**C**,**G**,**K**) Calculated SO_2_ dynamics overlaid with grayscale US B-mode images. (**D**,**H**,**L**) C-scan image of SO_2_ dynamics overlaid with US. Rotational 3D images can be seen in Supplementary Materials Video S1.

**Figure 6:**
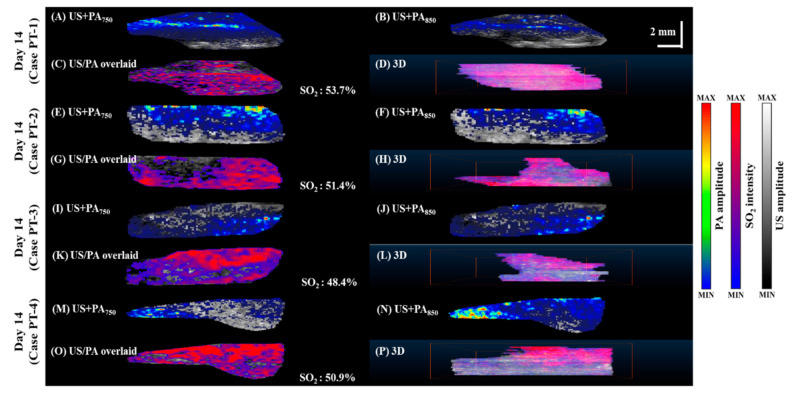
Relative SO_2_ levels in pancreatic tumor in mice imaged 14 days after the tumor cell injection. (**A**,**E**,**I**,**M**) PA images acquired at 750 nm overlaid with grayscale US B-mode images. (**B**,**F**,**J**,**N**) PA images acquired at 850 nm overlaid with grayscale US B-mode images. (**C**,**G**,**K**,**O**) SO_2_ was calculated as the percentage of oxygenated hemoglobin relative to total hemoglobin based on dual-wavelength PA imaging at 750 and 850 nm and overlaid with grayscale US B-scan images. (**D**,**H**,**L**,**P**) C-scan of SO_2_ dynamics. Rotational 3D images can be seen in Supplementary Materials Video S2.

**Figure 7:**
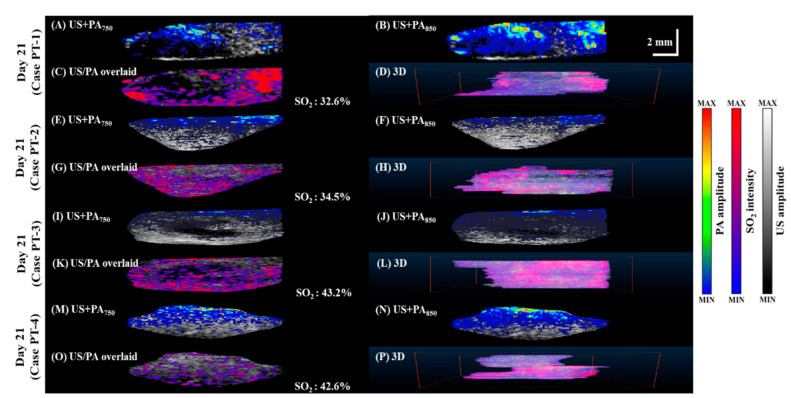
Relative SO_2_ levels in pancreatic tumor in mice imaged 21 days after tumor cell injection. (**A**,**E**,**I**,**M**) PA images acquired at 750 nm overlaid with grayscale US B-mode images. (**B**,**F**,**J**,**N**) PA images acquired at 850 nm overlaid with grayscale US B-mode images. (**C**,**G**,**K**,**O**) SO_2_ was calculated as the percentage of oxygenated hemoglobin relative to total hemoglobin based on dual-wavelength PA imaging at 750 and 850 nm and overlaid with grayscale US B-scan images. (**D**,**H**,**L**,**P**) C-scan of SO_2_ dynamics. Rotational 3D images can be seen in Supplementary Materials Video S3.

To compare tissue oxygenation in normal vs. tumor-bearing mice, we imaged SO_2_ levels in the pancreas of normal mice (Figure 5), mice 14 days after tumor implantation (Figure 6), and mice 21 days after tumor implantation (Figure 7). PA B-scan images acquired at 750 nm (Figure 5A,E,I, Figure 6A,E,I,M and Figure 7A,E,I,M) and 850 nm (Figure 5B,F,J, Figure 6B,F,J,N and Figure 7B,F,J,N) were used to calculate SO_2_ (Figure 5C,G,K, Figure 6C,G,K,O and Figure 7C,G,K,O). The 3D C-scan images (Figure 5D,H,L, Figure 6D,H,L,P and Figure 7D,H,L,P) were generated by combining B-scan images. The corresponding rotational 3D images can be seen in Supplementary Materials Videos S1–S3.

**Figure 8:**
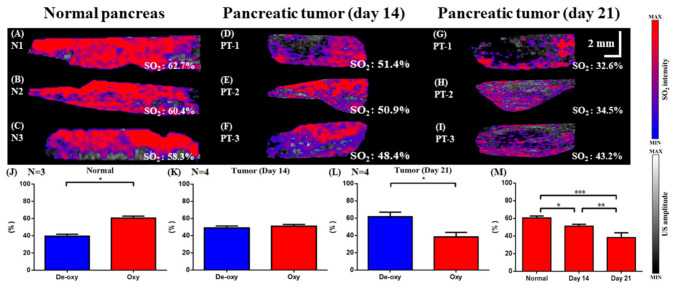
Comparison of the relative SO_2_ levels in the pancreas of normal and tumor-bearing mice on day 14 and day 21. (**A**–**C**) Representative images of SO_2_ levels in normal mice. (**D**–**F**) Representative images of SO_2_ levels in tumor-bearing mice on day 14 after the tumor cell injection and on day 21 (**G**–**I**). (**J**) Quantification of the levels of deoxy vs. oxyhemoglobin in normal and tumor-bearing mice on day 14 (**K**) and day 21 (**L**). (**M**) Comparison of tissue SO_2_ in normal, day 14, and day 21 tumors (* *p* < 0.05, ** *p* < 0.01, *** *p* < 0.001).

Figure 8 shows a comparison of the relative SO_2_ levels in the pancreas of normal and tumor-bearing mice on day 14 and day 21. Representative images selected from C-scan imaging of SO_2_ levels in normal, day 14, and day 21 mice are shown in Figure 8A–C,D–F, and G–I, respectively. Quantification of the levels of deoxy vs. oxyhemoglobin are shown in Figure 8J–L. In normal mice, the percentage of oxyhemoglobin was significantly greater than the percentage of deoxyhemoglobin (Figure 8J). In contrast, the deoxyhemoglobin levels were significantly higher in day 21 tumor-bearing mice (Figure 8L), suggesting a hypoxic environment. Comparison of tissue SO_2_ levels between the three groups of mice (Figure 8M) shows significant differences, and a downward trend is seen from normal to day 14 to day 21.

Currently, most preclinical PA imaging systems are used in preclinical studies to assess the tumor microenvironment, hypoxia, angiogenesis, and metastasis [46]. Our device for measuring real-time PA signals uses hemoglobin as an endogenous contrast agent with absorption wavelengths of 750 and 850 nm to detect SO_2_ and vascular blood volume at a wavelength of 800 nm [36] in pancreatic tumors. Previously, hemoglobin was used in PA imaging of brain, breast, and prostate malignancies [4,32,33]; however, data on the dynamic changes in pancreatic cancer models in vivo are limited. Hypoxia within solid tumors is often responsible for the failure of chemotherapy or radiotherapy. The generation of a hypoxic environment and activation of its main effector, hypoxia-inducible factor-1 (HIF-1), leads to tumor progression in advanced cancer [44].

### 3.5. PA Imaging of Pancreatic Tumor Hypoxia Dynamics Is Confirmed by IHC Staining for Pimonidazole

**Figure 9:**
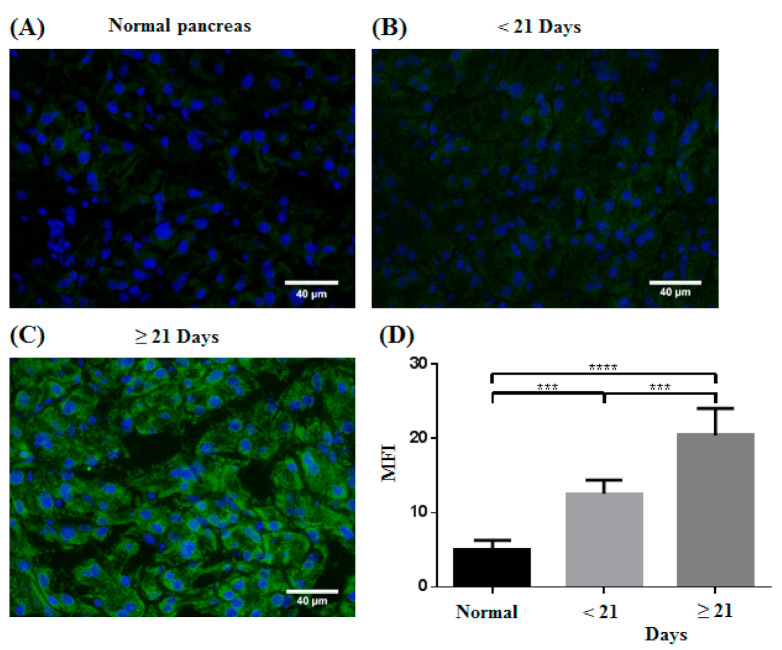
(**A**–**C**) Representative images of IHC staining for hypoxia using pimonidazole in the samples of normal pancreas and pancreatic tumors on various days after the tumor cell injection. The nucleus is presented in blue, and areas that stained positive for pimonidazole are shown in green. (**D**) Quantification of the MFI of pimonidazole staining. (n = 6 for normal pancreas, n = 5 for < 21 days, n = 4 for ≥ 21 days; *** *p* ≤ 0.001, **** *p* ≤ 0.0001).

To confirm the results of the PA measurements of SO_2_ in pancreatic tumors, IHC staining for hypoxia using pimonidazole was used to compare the samples of the normal pancreas (n = 6), pancreatic tumors < 21 days after the injection (n = 5) and pancreatic tumors ≥ 21 days after the injection (n = 5); representative images of the samples after pimonidazole staining are shown in Figure 9A–C. Quantification of MFI in Figure 9D shows a significant increase in pimonidazole staining in the pancreatic tumor samples compared with that in the normal pancreatic samples. Tumors ≥ 21 days after the injection also exhibited significantly greater staining. This increase in hypoxia is in agreement with the results of the dual-wavelength PA measurement, which showed a decrease in tissue SO_2_ in tumors ≥ 21 days after injection compared with tumors < 21 days after the injection (unpaired *t*-test, *p* ≤ 0.001). There was high agreement between the histological evaluation of hypoxia in the tumor microenvironment and qualitative and quantitative results of the PA signal analysis, indicating that our device is valid for real-time hypoxia assessment.

Nanodrugs, such as nab-paclitaxel and nanoliposomal irinotecan, have been widely used in the treatment of pancreatic adenocarcinoma [47]. Nanoparticles responsible for enhancing oxygen levels within the tumor and sensitizing hypoxic areas due to the suppression of hypoxia-inducing factors can lead to a decrease in tumor hypoxia and thus enhance the therapeutic efficacy of chemotherapy and immunotherapy [48,49,50,51]. In future studies, we will test therapeutic drugs for pancreatic cancer by comparing a parental drug and nanoparticle-based formulations and monitoring the dynamic changes in hypoxia in tumor tissue using our non-invasive real-time dual-modality PA/US system. These efforts will lead to improvements in the design of future clinical trials, expand the perspective to include the molecular and histological implications of novel treatment paradigms, and ultimately change the lives of patients.

## 4. Discussion

In this study, we present a dual-modality US/PA imaging system that can be used to assess tissue oxygenation in vivo. We initially tested our system with ink-filled phantoms to mimic the in vivo vasculature. Then, we demonstrated that the system could image the vasculature in rat skin. To assess whether our system can quantify tissue oxygenation, the next step involved a controlled hypoxia challenge. The results showed that our system is comparable to pulse oximetry, while having the advantage of presenting spatial information. Then, we imaged the pancreas of normal and tumor-bearing mice. The results demonstrated that our dual-wavelength PA technique, based on the measurement of oxygenated and deoxygenated hemoglobin, yields reliable real-time hypoxia data in vivo in an orthotopic pancreatic tumor mouse model. The PA SO_2_ measurements were confirmed by IHC staining for hypoxia, thus validating the applicability of PA for monitoring the functional aspects of tumorigenesis. A previous study by Nania et al. also demonstrated correlation between PA measurement of SO_2_ and IHC staining for hypoxia in mouse tumor models [52]. The developed dual-wavelength PA technique can be widely used because it does not require an exogenous contrast agent. The results of this study indicate the feasibility of real-time dual-wavelength PA imaging of a preclinical pancreatic tumor mouse model to assess tissue hypoxia. In future studies, the developed dual-wavelength PA imaging system can potentially be used to investigate the relationships between hemodynamics and specific biomarkers associated with tumor progression and to evaluate the therapeutic efficacy of treatment.

## Data Availability

Data will be provided on request through the corresponding author (Lun-De Liao) of this article.

## Supplementary Materials

The following are available online at https://www.mdpi.com/article/10.3390/mi12060668/s1, Video S1 for Figure 5, Video S2 for Figure 6 and Video S3 for Figure 7.
